# Clean Electrochemical Synthesis of Pd–Pt Bimetallic Dendrites with High Electrocatalytic Performance for the Oxidation of Formic Acid

**DOI:** 10.3390/ma15041554

**Published:** 2022-02-18

**Authors:** Jie Liu, Fangchao Li, Cheng Zhong, Wenbin Hu

**Affiliations:** 1 Key Laboratory of Advanced Ceramics and Machining Technology (Ministry of Education), School of Materials Science and Engineering, Tianjin University, Tianjin 300072, China; jieliu0109@tju.edu.cn (J.L.); lfc2018208170@tju.edu.cn (F.L.); wbhu@tju.edu.cn (W.H.); 2 Tianjin Key Laboratory of Composite and Functional Materials, School of Materials Science and Engineering, Tianjin University, Tianjin 300072, China; 3 Joint School of National University of Singapore and Tianjin University, International Campus of Tianjin University, Binhai New City, Fuzhou 350207, China

**Keywords:** electrochemical synthesis, Pd–Pt dendrites, formic acid oxidation, high electrocatalytic performance

## Abstract

Pd–Pt bimetallic catalysts with a dendritic morphology were in situ synthesized on the surface of a carbon paper via the facile and surfactant-free two step electrochemical method. The effects of the frequency and modification time of the periodic square-wave potential (PSWP) on the morphology of the Pd–Pt bimetallic catalysts were investigated. The obtained Pd–Pt bimetallic catalysts with a dendritic morphology displayed an enhanced catalytic activity of 0.77 A mg^−1^, almost 2.5 times that of the commercial Pd/C catalyst reported in the literature (0.31 A mg^−1^) in acidic media. The enhanced catalytic activity of the Pd–Pt bimetallic catalysts with a dendritic morphology towards formic acid oxidation reaction (FAOR) was not only attributed to the large number of atomic defects at the edges of dendrites, but also ascribed to the high utilization of active sites resulting from the “clean” electrochemical preparation method. Besides, during chronoamperometric testing, the current density of the dendritic Pd–Pt bimetallic catalysts for a period of 3000 s was 0.08 A mg^−1^, even four times that of the commercial Pd/C catalyst reported in the literature (about 0.02 A mg^−1^).

## 1. Introduction

Noble metals are widely used in various important catalytic or electrocatalytic reactions in the fields of energy conversion/storage as well as water pollution such as hydrogen production [1], reduction of oxygen [2], oxidation of organic or inorganic small molecule [3], fuel cells [4], and degradation of organic dyes in water [5]. Among these important applications, fuel cells have been receiving extensive attention due to their ability to directly convert the chemical energy of small-molecule fuel oxidation into electricity [6]. Direct formic acid fuel cells (DFAFCs) are believed to be a promising power generation system owing to their reasonable power density, high electromotive force, and limited fuel crossover [7,8]. As a key component of DFAFCs, electrocatalysts towards formic acid oxidation reaction (FAOR) play a vital role in the development of DFAFCs. Recently, noble metal palladium (Pd) has drawn intensive attention due to its high catalytic activity for FAOR and better resistance to CO poisoning than Pt [9,10,11,12]. However, the Pd catalyst is prone to dissolution in acidic media during catalytic reactions, hampering its commercial application [13]. In addition, there is still much room for improvement in the catalytic activity of the Pd catalyst. Therefore, it is particularly eager to develop a highly active and durable catalyst towards FAOR.

It is widely accepted the oxidation of formic acid involves two mechanisms, i.e., the direct oxidation pathway (HCOOH → CO_2_ + 2H^+^ + 2e^−^) and the CO oxidation pathway (HCOOH → CO_ads_ + H_2_O → CO_2_ + 2H^+^ + 2e^−^) [14]. In the direct oxidation pathway, HCOOH molecules directly dehydrogenate to form CO_2_ via one or more active intermediates. In the CO oxidation pathway, HCOOH molecules dehydrate to produce CO which depends on the applied potential. The generated CO may be further oxidized to CO_2_ or poison the catalyst. Up to date, several strategies have been developed to improve the electrocatalytic properties of Pd. The previous literature has reported that alloys of Pd with other metals (such as Cu [9], Pt [15], Co [16], Ag [17], Au [18], Sn [19], Ni [20], Rh [21], Zn [22], Pb [23], Cr [24], and Ir [25]) can not only enhance catalytic activity, but also improve the corrosion resistance of Pd. In particular in Pd–Pt alloys, the electronegativities and bulk Wigner–Seitz radii of Pt and Pd are similar [26]. The alloying of Pt and Pd will produce a synergistic electronic effect [27]. This effect favors formic acid oxidation via the direct oxidation pathway. Additionally, Pt also exhibits high stability in acidic media due to its chemically inert property [28].

It is generally accepted the morphology of catalysts plays a crucial role in enhancing the catalytic activity. A great deal of work has focused on the synthesis of bimetallic Pt–Pd catalysts with various morphologies. For instance, Guo et al. [15] synthesized three-dimensional (3D) dendritic Pt-on-Pd bimetals on graphene sheets via a facile wet-chemical approach and found that Pt–Pd bimetallic nanodendrites/graphene hybrids exhibited high catalytic activity for the oxidation of methanol. Yuan et al. [29] prepared Pd–Pt random alloy nanocubes in an aqueous solution containing KBr, polyvinyl pyrrolidone, and sodium lauryl sulfate with PdCl_2_ and K_2_PtCl_6_ as precursors. Zhang et al. [30] reported different shapes of Pd–Pt alloys by a solvothermal process, such as flowers, dendrites, bars, cubes, and concave cubes, and concluded that the obtained Pd–Pt alloys had an enhanced catalytic activity and CO tolerance towards FAOR. Lu et al. [31] fabricated a reduced graphene oxide/Pt–Pd alloy nanocubes by a facile hydrothermal method. However, the synthesis of most Pd–Pt catalysts with various morphologies commonly uses organic additives, thus requiring additional removal processes of additives. Otherwise, incomplete removal of organic additives limits the active sites of the catalyst and thus negatively affects its performance. Additionally, during conventional electrode preparation, the powder catalysts have to be transferred to the surface of the electrode, which inevitably requires the use of conductive additives and binders. The use of conductive additives and binders will lead to a reduction in the active sites of the catalyst. Therefore, it is highly desired to develop a facile and surfactant-free route to synthesize Pd–Pt nanocatalysts. The electrochemical synthesis method is considered to be an effective catalyst preparation method because of its simplicity, low cost, easy operation, and high purity of the product [32]. However, conventional electrochemical synthesis techniques have limited control parameters, making it difficult to effectively control the morphology of the catalyst. The combination of different modes of electrochemical technologies can effectively expand the range of its control parameters and is an effective method to realize the control of the catalyst morphology. For example, Tian et al. [33,34] synthesized monometallic Pt and Pd tetrahexahedral nanocrystals by the electrochemical technology, which showed a high electrocatalytic activity for the oxidation of formic acid and ethanol. The dendritic Pt and highly dispersed Pd particles also synthesized by a similar electrochemical method and displayed a high electrocatalytic activity [35,36]. However, there are few reports on the synthesis of morphology-controlled Pd–Pt bimetallic catalysts via a clean electrochemical approach. The research on the effect of electrochemical parameters on the structure and morphology of Pd–Pt bimetallic catalysts is very limited. The intrinsic relationship between the microstructure of Pd–Pt bimetallic catalysts and its macroscopic catalytic performance also needs to be further illustrated.

Herein, Pd–Pt bimetallic catalysts with a dendritic morphology were in situ synthesized on the surface of a carbon paper by a facile and clean electrochemical method. Pure Pd particles were firstly electrodeposited on the surface of the carbon paper, and then Pd–Pt bimetallic catalysts with a dendritic morphology were obtained by the periodic square-wave potential (PSWP) treatment of pure Pd particles in an aqueous solution of 0.5 M H_2_SO_4_ and 5 mM PdCl_2_. The effects of the frequency and treatment time of the PSWP on the morphology of the bimetallic Pd–Pt catalysts were systematically investigated. The obtained Pd–Pt bimetallic catalyst with a dendritic morphology displayed an outstanding catalytic activity (0.77 A mg^−1^) and a high stability towards FAOR.

## 2. Experimental

### 2.1. Reagents and Materials

Palladium (II) chloride (PdCl_2_; Adamas Reagent Co., Ltd., Shanghai, China), chloroplatinic acid hexahydrate (H_2_PtCl_6_·6H_2_O; Shanghai Aladdin Biochemical Technology Co., Ltd., Shanghai, China), sulfuric acid (H_2_SO_4_; Jiangtian Chemical Technology Co., Ltd., Tianjin, China), formic acid, and anhydrous ethanol (Yuanli Chemical Technology Co., Ltd., Tianjin, China) were of analytical reagent grade. A carbon paper (TGP-H-060) and commercial 10 wt % Pd/C catalysts were purchased from Toray (Toray Industries, Tokyo, Japan) and Shanghai Aladdin Biochemical Technology Co., Ltd. (Shanghai, China), respectively. The deionized water was produced with a Millipore Milli-Q system (18.2 MΩ cm).

### 2.2. Electrochemical Fabrication of Pd Particles and Pd–Pt Bimetallic Catalysts

Firstly, prior to the electrodeposition of Pd particles, the carbon paper was ultrasonically cleaned with anhydrous ethanol, acetone, and deionized water for 30 min separately. Secondly, Pd particles was electrochemically deposited on the surface of the carbon paper at −0.15 mA cm^–2^ for 45 min in an aqueous solution of 5 mM PdCl_2_ and 0.5 M H_2_SO_4_, using a conventional electrochemical cell with a three-electrode system (Ivium Stat, Ivium Technologies, Eindhoven, Netherlands). The cleaned carbon paper served as the working electrode. A platinum plate electrode and a mercurous sulfate electrode (MSE) were used as the counter electrode and the reference electrode, respectively. At last, based on the obtained pure Pd particles, a Pd–Pt bimetallic catalysts/carbon paper electrode was finally synthesized in a two-electrode system by PSWP (NF BP4610, NF Corporation, Yokohama Japan) with different frequencies (10 Hz, 50 Hz, and 90 Hz) and modification times (1 h, 2 h, and 4 h) in an aqueous solution of 0.1 mM H_2_PtCl_6_ and 1 M H_2_SO_4_. Correspondingly, the obtained Pd–Pt samples were denoted as PdPts–10 Hz, PdPts–50 Hz (4 H), PdPts–90 Hz, PdPts–1 H, and PdPts–2 H, respectively. The upper and lower limit potentials of the PSWP were 0.6 V and −3.2 V, respectively.

### 2.3. Characterization of Pd Particles and Pd–Pt Bimetallic Catalysts

The surface morphologies of the Pd particles and the Pt−Pd bimetallic catalysts were investigated by a field-emission scanning electron microscope (Hitachi S-4800, Hitachi, Tokyo, Japan). The structure and composition of the Pt−Pd bimetallic catalysts were investigated by a transmission electron microscope (JEOL 2100F, JELO Ltd., Tokyo Japan) equipped with energy-dispersive X-ray (EDX) analysis. The phase and the crystallinity of the Pd–Pt bimetallic catalysts were analyzed by X-ray diffraction (XRD; Bruker D8 Advanced, Billerica, MA, USA) with CuK_α_ radiation (λ = 1.5418 Å). The loading amounts of Pd and Pt particles were obtained by inductively coupled plasma-mass spectrometry (ICP-MS; Agilent 7700, Agilent Technologies, Santa Clara, CA, US).

### 2.4. Electrochemical Test

All electrochemical tests were performed in a conventional three-electrode system (Ivium Stat, Ivium Technologies, Eindhoven, Netherlands). The obtained Pd–Pt bimetallic catalyst/carbon paper electrode was used as the working electrode. A platinum plate and a saturated calomel electrode (SCE) served as the counter and the reference electrodes, respectively. The cyclic voltammetry (CV) curves of the obtained Pd–Pt bimetallic catalysts were tested in a N_2_-saturated aqueous solution of 0.5 M H_2_SO_4_ at a scan rate of 50 mV s^−1^. The voltammetric curves of the obtained Pd–Pt bimetallic catalysts towards FAOR were recorded in an aqueous solution of 0.5 M H_2_SO_4_ and 0.5 M HCOOH at a scan rate of 50 mV s^−1^. The chronoamperometry curves of the obtained Pd–Pt bimetallic catalysts were recorded in an aqueous solution of 0.5 M H_2_SO_4_ and 0.5 M HCOOH at 0.15 V for 3000 s.

## 3. Results and Discussion

**Figure 1:**
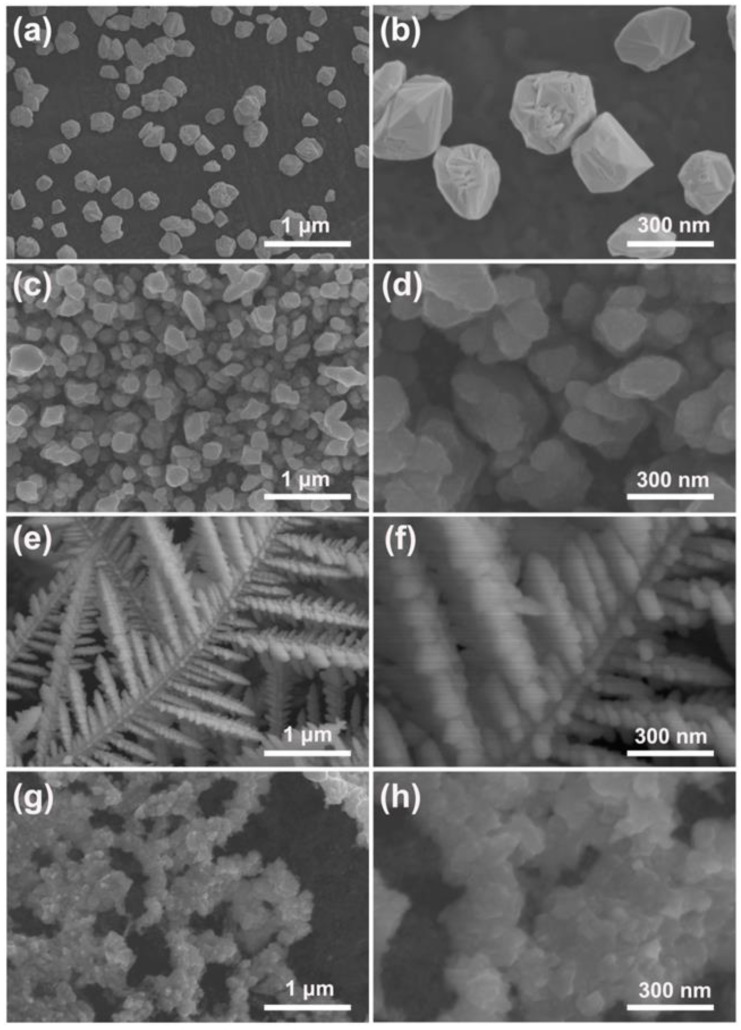
SEM images of pure Pd particles (**a**) and the obtained Pd–Pt bimetallic catalysts modified by the periodic square-wave potential (PSWP) with the frequencies of 90 Hz (**c**), 50 Hz (**e**), 10 Hz (**g**) for 4 h in a solution of 0.1 mM H_2_PtCl_6_ + 1 M H_2_SO_4_. (**b**,**d**,**f**,**h**) are the high-magnification SEM images of the corresponding catalysts.

Figure 1 displays the SEM images of the pure Pd particles and the Pd–Pt bimetallic catalysts with various morphologies fabricated by PSWP modification with different frequencies in an aqueous solution containing a Pt precursor. The pure Pd particles were electrodeposited on the surface of the carbon paper (Figure 1a), and the average particle size was about 270 nm (Figure 1b). A large number of Pd–Pt particles with a relatively smooth surface were electrodeposited on the surface of the carbon paper, after the pure Pd particles were treated by the PSWP with a frequency of 90 Hz (Figure 1c,d). The size of the Pd–Pt particles was similar to that of the pure Pd particles (Figure 1d). When the modification frequency of the PSWP decreased to 50 Hz, the obtained Pd–Pt bimetallic catalysts showed a well-defined dendritic morphology (Figure 1e). A lot of long secondary dendrites grew on the trunk of the dendrite of the Pd–Pt bimetallic catalysts, and the short tertiary dendrites were also formed on the secondary dendrite arms (Figure 1e,f). As the frequency of the PSWP further reduced to 10 Hz, the Pd–Pt bimetallic catalysts showed an agglomerated morphology with a rough surface (Figure 1g,h). Obviously, the frequency of the PSWP had a significant effect on the morphology of the Pd–Pt bimetallic catalysts. This may be related to the process of adsorption/desorption of oxygen on the surface of Pd and Pt metals during PSWP modification [37]. The oxidation processes of the Pd and Pt surfaces were affected by the upper limit potential of the PSWP, and the dynamic adsorption/desorption process of oxygen on the Pd and Pt surfaces affected the dissolution of Pd and Pt. The lower limit potential of the PSWP was responsible for the deposition of Pd and Pt. When the frequency of the PSWP was high (90 Hz), the square-wave period of the Pd particle surface was relatively short, and thus the modification effect on the surface morphology of Pd particles was limited. As a result, many Pd–Pt particles with a relatively smooth surface were formed (Figure 1c,d), and their morphology was similar to that of pure Pd particles (Figure 1a,b). As the frequency of the PSWP decreased to 50 Hz, the square-wave period increased, resulting in a long period of the dissolution and deposition of metal atoms on the surface of Pd particles. The long-period deposition process caused the diffusion-limited growth of metal ions near the electrode surface. These metal ions tended to diffuse towards the tip of the electrode surface, which finally led to the formation of Pd–Pt bimetallic catalysts with a dendritic morphology (Figure 1e,f). When the frequency of the PSWP reduced to the smallest frequency, i.e., 10 Hz, the PSWP period was longer. The formed dendrites were dissolved, since the dissolution caused by the upper limit potential played a dominant role during the long square-wave period. Consequently, the Pd–Pt bimetallic catalysts displayed an agglomerated morphology with a rough surface (Figure 1g,h).

**Figure 2:**
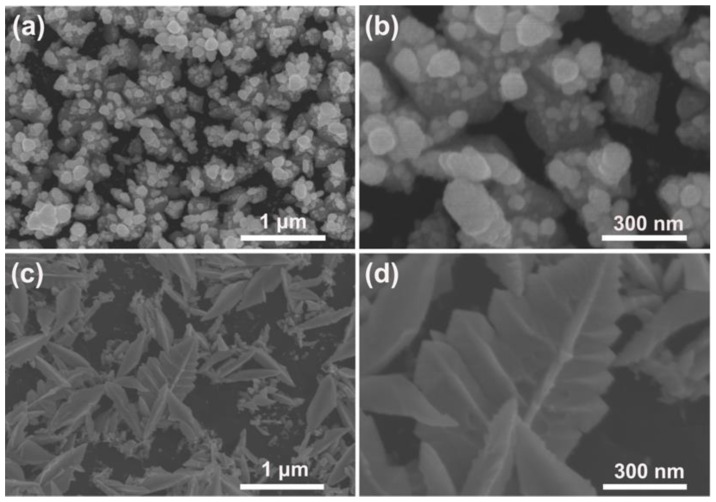
SEM images of the obtained Pd–Pt bimetallic catalysts modified by the PSWP with a frequency of 50 Hz for 1 h (**a**) and 2 h (**c**) in a solution of 0.1 mM H_2_PtCl_6_ + 1 M H_2_SO_4_. (**b**,**d**) are the high-magnification SEM images of the corresponding Pd–Pt bimetallic catalysts.

To further gain insight into the formation mechanism of the Pd–Pt bimetallic catalysts with a dendritic morphology, the surface morphology of the Pd–Pt bimetallic catalysts as a function of the modification time of the PSWP was investigated. Figure 2 shows the SEM images of the Pd–Pt bimetallic catalysts obtained by the PSWP modification with different times. As the modification time increased from 1 h to 4 h, the morphology of the modified Pd–Pt bimetallic catalysts evolved from the agglomerated particles (Figure 2a,b), leaf-like catalysts (Figure 2c,d) to dendritic catalysts (Figure 1e,f). During modification, in the early stage (modification time of 1 h) of the formation of the Pd–Pt bimetallic catalysts, the short modification time had limited effect on the morphology of the Pd–Pt bimetallic catalysts. Only the Pd–Pt bimetallic catalysts with an agglomerated morphology were observed (Figure 2a,b). When the modification time was extended to 2 h, the diffusion of metal ions near the electrode surface was limited, and the metal ions diffused toward the tip of the electrode surface during the deposition process. As a result, the leaf-like catalysts with a protruding texture were formed (Figure 2c,d). As the modification time was further extended to 4 h, the leaf-like catalysts were selectively dissolved, only leaving the protruding texture part. Finally, the Pd–Pt bimetallic catalysts displayed a well-defined dendritic morphology (Figure 1e,f).

**Figure 3:**
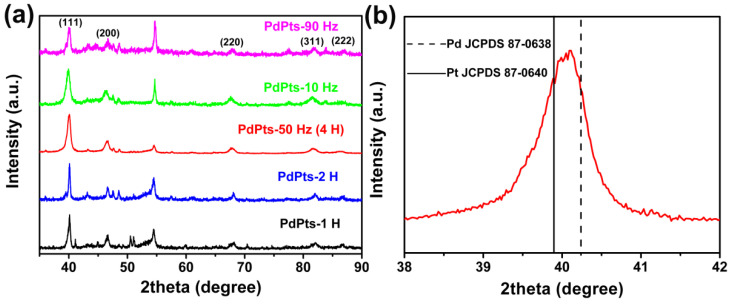
(**a**) X-ray diffraction (XRD) patterns of the obtained Pd–Pt bimetallic catalysts; (**b**) the enlarged XRD spectrum of the Pd–Pt bimetallic catalysts with a dendritic morphology in the 2θ range of 38−42°.

Figure 3 displays the XRD spectra of the Pd–Pt catalysts. All the catalysts showed the distinct characteristic diffraction peaks at about 40.1°, 46.6°, 67.9°, 81.7°, and 86.7°, which corresponded to the (111), (200), (220), (311), and (222) lattice planes of the Pd–Pt bimetallic catalysts, respectively (Figure 3a) [38]. This indicated that the obtained Pd–Pt bimetallic catalysts possessed a polycrystalline structure. Figure 3b shows the enlarged (111) peaks of the Pd–Pt bimetallic catalysts with a dendritic morphology. It was found that the (111) peak of the Pd–Pt bimetallic catalysts with a dendritic morphology shifted to the position between the (111) peaks of monometallic Pt (JCPDS 87–0640) and Pd (JCPDS 87–0638). This phenomenon was attributed to the substitution of Pd atoms with Pt in the lattice, which resulted in the expansion of the face-centered cubic lattice [38], indicating the successful formation of Pd–Pt alloy.

**Figure 4:**
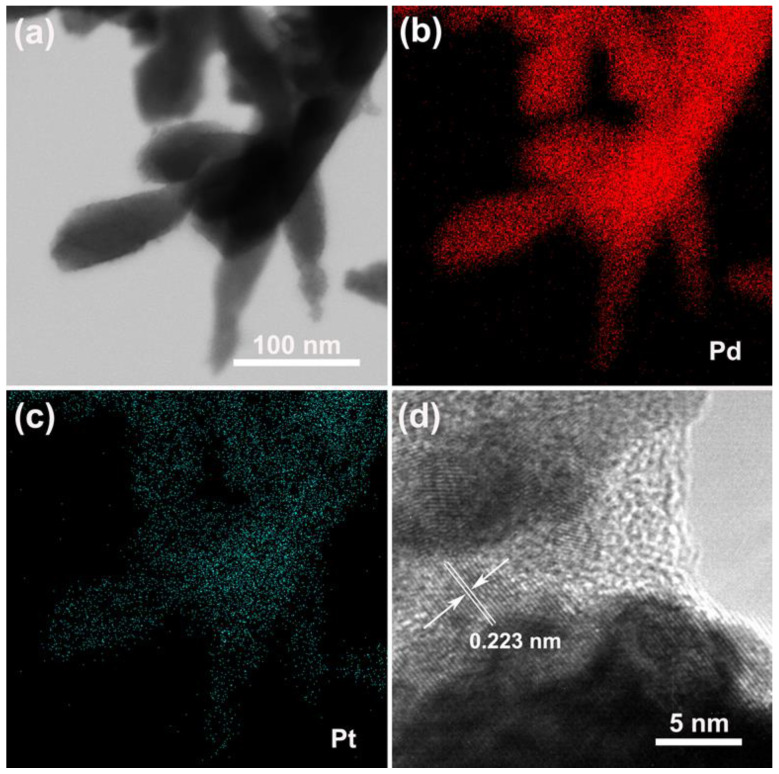
(**a**) TEM image of Pd–Pt dendrites. The elemental mapping images of Pd (**b**) and Pt (**c**) of Pd–Pt dendrites. (**d**) High-resolution TEM (HRTEM) image of Pd–Pt dendrites.

To obtain the detailed structural information of the Pd–Pt bimetallic catalysts with a dendritic morphology, the TEM analysis was conducted. Figure 4a displays the TEM images of the obtained Pd–Pt bimetallic catalysts with a dendritic morphology. It was observed that the long nanothorns grew on the tip of the Pd–Pt dendrites. The length of the nanothorns was about 100 nm. This is coincident with the observed SEM results (Figure 1e,f). Figure 4b,c shows the elemental mapping images of the corresponding Pd–Pt dendrites. The Pd–Pt dendries were composed of Pd (Figure 4b) and Pt (Figure 4c) elements, and these elements were uniformly distributed on the surface of Pd–Pt dendrites. Figure 4d shows the high-resolution TEM (HRTEM) image of the Pd–Pt dendrites. The well-defined lattice fringes of the Pd–Pt dendrites were observed, indicating the high crystallinity of the Pd–Pt dendrites. The interplanar spacing of the Pd–Pt dendrites was 0.223 nm, which matched with the (111) facet of the Pd–Pt phase (confirmed by XRD; Figure 3) [6].

**Figure 5:**
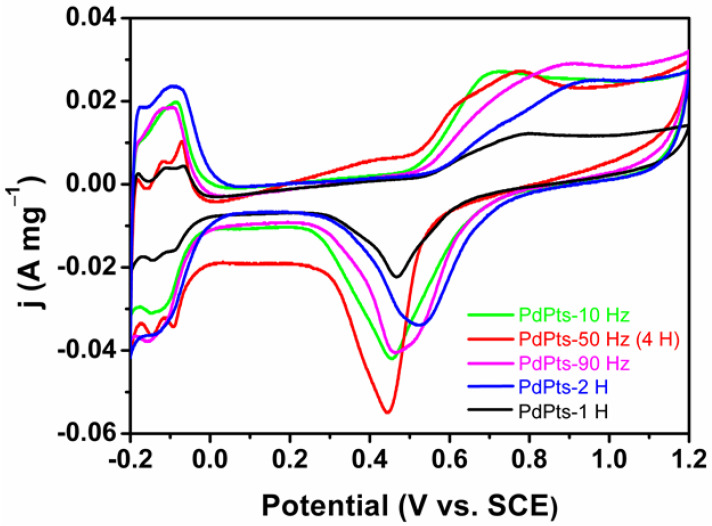
Cyclic voltammetry curves of the obtained Pd–Pt bimetallic catalysts tested in a 0.5 M H_2_SO_4_ solution at a scan rate of 50 mV s^−1^, normalized by the Pd–Pt mass.

Figure 5 shows the CV profiles of the Pd–Pt bimetallic catalysts tested in a N_2_-saturated 0.5 M H_2_SO_4_ solution. All the CV curves displayed the similar voltammetric features to Pd–Pt polycrystalline. These CV curves exhibited three typical potential regions including the hydrogen adsorption/desorption (–0.20 V to 0.04 V (vs. SCE)), the electric double layer (0.04 V to 0.50 V (vs. SCE)), and the formation/reduction of Pt/Pd oxides (0.50 V to 1.20 V (vs. SCE)) [39]. The multiple peaks of the hydrogen adsorption/desorption of the Pd–Pt bimetallic catalysts indicated that the Pd–Pt bimetallic catalysts possessed a well-developed polycrystalline structure [40,41], which coincided with the XRD results. The electrochemically active surface area (ECSA) can be estimated by integrating a columbic charge associated with reduction peaks of Pd/Pt oxides at about 0.47 V after electric double layer correction, assuming that the charge required for the reduction of the Pd/Pt oxides monolayer was 424 µC cm^−2^ [42]. The calculated ECSAs of PdPts–10 Hz, PdPts–50 Hz (4 H), PdPts–90 Hz, PdPts–1 H, and PdPts–2 H were 34.43 m^2^ g^−1^, 28.30 m^2^ g^−1^, 33.96 m^2^ g^−1^, 14.15 m^2^ g^−1^, and 31.60 m^2^ g^−1^, respectively. The relatively small specific ECSA of the Pt−Pd bimetallic catalysts with a dendritic morphology can be ascribed to the large dendrite size. This is consistent with the observed SEM results (Figure 1).

**Figure 6:**
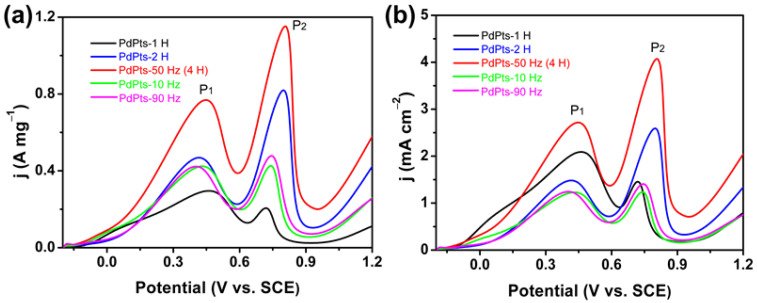
Voltammetric curves of the obtained Pd–Pt bimetallic catalysts in an aqueous solution of 0.5 M H_2_SO_4_ and 0.5 M HCOOH at a scan rate of 50 mV s^−1^, normalized by the Pd–Pt mass (**a**) and the Pd–Pt electrochemically active surface area (ECSA) (**b**).

To evaluate the catalytic activity of the Pd–Pt catalysts, the voltammetry tests of Pd–Pt catalysts towards FAOR were conducted. Figure 6 shows the voltammetric curves of the Pd–Pt bimetallic catalysts towards FAOR. Two well-defined current peaks P_1_ and P_2_ appeared at about 0.44 V (vs. SCE) and 0.78 V (vs. SCE) (Figure 6a), corresponding to the direct oxidation of formic acid to CO_2_ and oxidation of adsorbed CO generated by dehydration of formic acid, respectively [7,14]. The Pd–Pt bimetallic catalysts with a dendritic morphology (PdPt–50 Hz (4 H)) exhibited the highest mass activity (P_1_, 0.77 A mg^−1^) among the obtained Pd–Pt bimetallic catalysts towards FAOR and was almost 2.5 times that of the commercial Pd/C catalyst (0.31 A mg^−1^) [36]. Furthermore, the Pd–Pt bimetallic catalysts with a dendritic morphology also displayed a higher mass activity compared with the reported Pd-based electrocatalysts towards FAOR (Table 1). Moreover, there was still much room for improvement in the mass activity of this catalyst by further reducing its particle size. Figure 6b shows the voltammetric curves of the Pd–Pt bimetallic catalysts normalized by the ECSA of Pd–Pt catalysts towards FAOR. The specific activity of the Pd–Pt bimetallic catalysts with a dendritic morphology was also much larger than those of the other obtained Pd–Pt bimetallic catalysts. The enhanced specific activity of the Pd–Pt bimetallic catalysts with a dendritic morphology towards FAOR can be ascribed to the large number of unsaturated atoms at the edges of dendrites (Figure 4d). During the preparation of conventional electrodes, the powder catalyst had to be mixed with polymer binders and conductive agents into a catalyst ink, and then the catalyst ink was transferred to the surface of a current collector. The introduction of polymer binders increased the interfacial resistance between the catalyst and the current collector. Besides, the physical transfer of the catalyst caused the agglomeration of the catalyst particles and thus reduced its effective catalytic active sites. These resulted in undesirable side effects on the catalytic activity of the catalyst. On contrary, the Pd–Pt bimetallic catalysts with a dendritic morphology were directly grown on the surface of the carbon paper, and the entire electrochemical synthesis process of the electrode did not involve the use of the binders and the transfer process of the catalyst. Consequently, the direct growth of dendritic Pd–Pt catalysts on the surface of the carbon paper could effectively reduce the interfacial resistance of electrode and maximize the utilization of the effective catalytic active sites of the catalyst, thereby improving the mass activity of the Pd–Pt bimetallic catalysts with a dendritic morphology. Therefore, the enhanced mass activity of the Pd–Pt catalysts with a dendritic morphology was not only attributed to the large number of atomic defects at the edges of dendrites, but also ascribed to the high utilization of active sites caused by the “clean” electrochemical preparation method. In addition, compared with commercial Pd/C catalysts, the electronic effects, caused by the proper downshift of the d-band center of Pd resulting from Pd alloying with Pt, contributed to accelerating the kinetic of FAOR, thus enhancing its catalytic activity [43,44].

**Figure 7:**
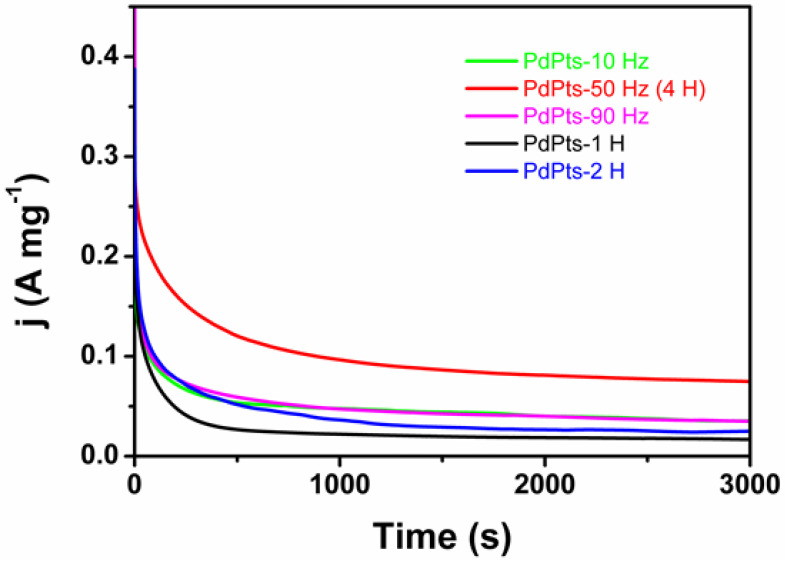
Chronoamperometry curves of the obtained Pd–Pt bimetallic catalysts recorded in an aqueous solution of 0.5 M H_2_SO_4_ and 0.5 M HCOOH at 0.15 V (vs. saturated calomel electrode (SCE)) for 3000 s.

To assess the stability of the Pd–Pt catalysts, the chronoamperometric testing of the Pd–Pt bimetallic catalysts towards FAOR was conducted. Figure 7 shows the chronoamperometric curves at 0.15 V (vs. SCE) for 3000 s. In the initial stage, a rapid drop of the current density was associated with electric double-layer charging [7]. Subsequently, a slow decay of the current density was observed, which was associated with surface poisoning by intermediates [7]. The current density of the Pd–Pt bimetallic catalysts with a dendritic morphology for a period of 3000 s was 0.08 A mg^−1^, which was higher than those of PdPts–10 Hz (0.04 A mg^−1^), PdPts–90 Hz (0.04 A mg^−1^), PdPts–1 H (0.02 A mg^−1^), and PdPts–2 H (0.03 A mg^−1^), even four times that of the commercial Pd/C catalyst reported in the literature (about 0.02 A mg^−1^) [36]. This indicated that the Pd–Pt bimetallic catalysts with a dendritic morphology possessed an outstanding catalytic activity and a high stability towards FAOR in an acid medium. Therefore, it is possible that the dendritic Pd–Pt catalyst directly electrodeposited on a carbon paper can be applied in direct formic acid fuel cells as a fuel cell anode, methanol fuel cells, and the degradation of organic dyes in water [5,54,55]. Additionally, since the catalyst can be continuously electrodeposited on the surface of a conductive substrate when the conductive substrate (such as carbon paper) is used as a “conveyor belt”, the electrochemical synthesis method in this work can realize the continuous industrial production of catalytic electrodes.

## 4. Conclusions

Pd–Pt catalysts with a dendritic morphology were in situ grown on the surface of a carbon paper via a facile and “green” two-step electrochemical method. The frequency of the PSWP had a significant effect on the morphology of the Pd–Pt bimetallic catalysts. Additionally, as the modification time increased, the morphology of the Pd–Pt bimetallic catalysts evolved from the agglomerated particles, leaf-like catalysts to dendritic catalysts. The obtained dendritic Pd–Pt catalysts displayed an outstanding catalytic activity (0.77 A mg^−1^) and a high stability towards FAOR. The improved catalytic activity of the Pd–Pt catalysts with a dendritic morphology can be ascribed to the high utilization of its active site and the improved specific activity related to its rough dendritic morphology. The dendritic Pd–Pt catalyst directly electrodeposited on a carbon paper possesses great potential to be applied in direct formic acid fuel cells as a fuel cell anode methanol fuel cells, and the degradation of organic dyes in water.

## Figures and Tables

**Table 1:** Comparison of the mass activity of the Pd–Pt bimetallic catalysts with a dendritic morphology prepared in this work with those of Pd-based electrocatalysts towards formic acid oxidation reaction (FAOR).

Catalyst	Test Protocol	Mass Activity (A mg^−1^)	Reference
Pd–Pt bimetallic catalysts with a dendritic morphology	0.5 M H_2_SO_4_ + 0.5 M HCOOH, 50 mV s^−1^	0.77	This work
Pd_1_Cu_3_/CNTs	0.5 M H_2_SO_4_ + 0.5 M HCOOH, 50 mV s^−1^	0.56	[45]
Pd/NS-G	0.5 M H_2_SO_4_ + 0.5 M HCOOH, 50 mV s^−1^	0.50	[46]
Pd_3_Pt half-shells	0.5 M H_2_SO_4_ + 0.5 M HCOOH, 50 mV s^−1^	0.32	[47]
Pd@graphene	0.5 M H_2_SO_4_ + 0.5 M HCOOH, 50 mV s^−1^	0.09	[48]
Pd/CN	0.5 M H_2_SO_4_ + 0.5 M HCOOH, 50 mV s^−1^	0.20	[49]
PdCuSn/CNFs	0.5 M H_2_SO_4_ + 0.5 M HCOOH, 50 mV s^−1^	0.53	[50]
Pt/Pd bimetallic nanotubes with a petal-like surface	0.5 M H_2_SO_4_ + 0.5 M HCOOH, 50 mV s^−1^	0.54	[51]
Pd_1_Ni_1_-NNs/RGO	0.5 M H_2_SO_4_ + 0.5 M HCOOH, 50 mV s^−1^	0.60	[52]
PdSnAg/C	0.5 M H_2_SO_4_ + 0.5 M HCOOH, 50 mV s^−1^	0.63	[53]
PdSn/C	0.5 M H_2_SO_4_ + 0.5 M HCOOH, 50 mV s^−1^	0.17	[53]
PdSnNi/C	0.5 M H_2_SO_4_ + 0.5 M HCOOH, 50 mV s^−1^	0.36	[53]
PdSnCo/C	0.5 M H_2_SO_4_ + 0.5 M HCOOH, 50 mV s^−1^	0.29	[53]

## Data Availability

Data are contained within the article.
